# Impact of Anaerobic Growth Conditions on Toxic Shock Syndrome Toxin-I Production by *Staphylococcus aureus*

**DOI:** 10.1155/S1064744996000440

**Published:** 1996

**Authors:** Max Lungren, Pongsakdi Chaisilwattana, Floyd C. Knoop, Gilles R. G. Monif

**Affiliations:** 1 Department of Medical Microbiology Creighton University School of Medicine Omaha NE USA; 2 Department of Obstetrics and Gynecology Creighton University School of Medicine 601 N. 30th Street Omaha NE 68131 USA

## Abstract

*Objective:* The impact of anaerobic growth conditions on the *Staphylococcus aureus* toxic shock syndrome toxin-1 (TSST-1) production was studied.

*Methods:* Ten strains of *S. aureus* derived from patients with toxic shock syndrome (TSS), 10 isolates of *S. aureus*, and documented TSST-1-producing strains recovered from patients with either staphylococcal septicemia or staphylococcal nongenital abscesses were grown under aerobic and anaerobic conditions. The bacterial growth was measured using optical density (OD) determinations at 520 nm. The toxin production was assayed using the TS-RPLA latex agglutination test.

*Results:* Both TSS and non-TSS strains of *S. aureus* grown under aerobic and anaerobic conditions exhibited comparable OD patterns of growth, and the levels of toxin production remained constant during the logarithmic phase. Toxin titers developed during the logarithmic growth phase and peaked after 24 h of incubation. When stationary-phase isolates grown initially under aerobic conditions were subjected to strict anaerobic conditions, subsequent toxin titers, compared with isolates grown in the continued presence of oxygen, were depressed 2-fold, peaking at a later time.

*Conclusions:* TSST-1 production is diminished under continued anaerobic conditions.


Infectious Diseases in Obstetrics and Gynecology 4:232–238 (1996)
© 1997 Wiley-Liss, Inc.

### Impact of Anaerobic Growth Conditions on Toxic Shock Syndrome Toxin-1 Production by *Staphylococcus aureus*

**Max Lungren, Pongsakdi Chaisilwattana, Floyd C. Knoop, and Gilles R.G. Monif**

*Departments of Medical Microbiology (M.L., F.C.K.) and Obstetrics and Gynecology (P.C., G.R.G.M.), Creighton University School of Medicine, Omaha, NE*

### Abstract

*Objective:* The impact of anaerobic growth conditions on the *Staphylococcus aureus* toxic shock syndrome toxin-1 (TSST-1) production was studied.

*Methods:* Ten strains of *S. aureus* derived from patients with toxic shock syndrome (TSS), 10 isolates of *S. aureus*, and documented TSST-1-producing strains recovered from patients with either staphylococcal septicemia or staphylococcal nongenital abscesses were grown under aerobic and anaerobic conditions. The bacterial growth was measured using optical density (OD) determinations at 520 nm. The toxin production was assayed using the TS-RPLA latex agglutination test.

*Results:* Both TSS and non-TSS strains of *S. aureus* grown under aerobic and anaerobic conditions exhibited comparable OD patterns of growth, and the levels of toxin production remained constant during the logarithmic phase. Toxin titers developed during the logarithmic growth phase and peaked after 24 h of incubation. When stationary-phase isolates grown initially under aerobic conditions were subjected to strict anaerobic conditions, subsequent toxin titers, compared with isolates grown in the continued presence of oxygen, were depressed 2-fold, peaking at a later time.

*Conclusions:* TSST-1 production is diminished under continued anaerobic conditions.
© 1997 Wiley-Liss, Inc.

Key words

Toxic shock syndrome, bacterial growth, tampons

Toxic shock syndrome (TSS) has been primarily a disease of tampon users at the time of menstruation.[1,2] Since the withdrawal of selected highly absorbent vaginal tampons and better-informed use of vaginal tampons, the prevalence of TSS has shifted back to clinical situations not associated with menstruation.[3,4] In obstetrics, the syndrome has been described following vaginal delivery, cesarean delivery, and spontaneous and therapeutic abortions. In the gynecologic patient, TSS has been documented as complicating surgical procedures such as tubal ligation, vaginal and abdominal hysterectomies, urethral and bladder suspensions, exploratory laparotomy, and therapy of extensive condyloma acuminatum.[5–8] TSS may occur in patients with mastitis, Bartholin's gland abscess, wound infection, or acute salpingitis. What did superabsorbent tampon utilization do to magnify the incidence of disease?

*Staphylococcus aureus*, the causative agent for TSS, and selected strains of *S. epidermidis* are the only genuses of bacteria with the capacity for being monoetiologic pathogens in both oxygen-rich and oxygen-deprived (abscess) situations. The volumetric expansion achieved by superabsorbent tampons introduced the possibility of the conversion of a

Address correspondence/reprint requests to Dr. Gilles R.G. Monif, Department of Obstetrics and Gynecology, Creighton University School of Medicine, 601 N. 30th Street, Suite 4700, Omaha, NE 68131.

Received March 20, 1996
Accepted July 17, 1996

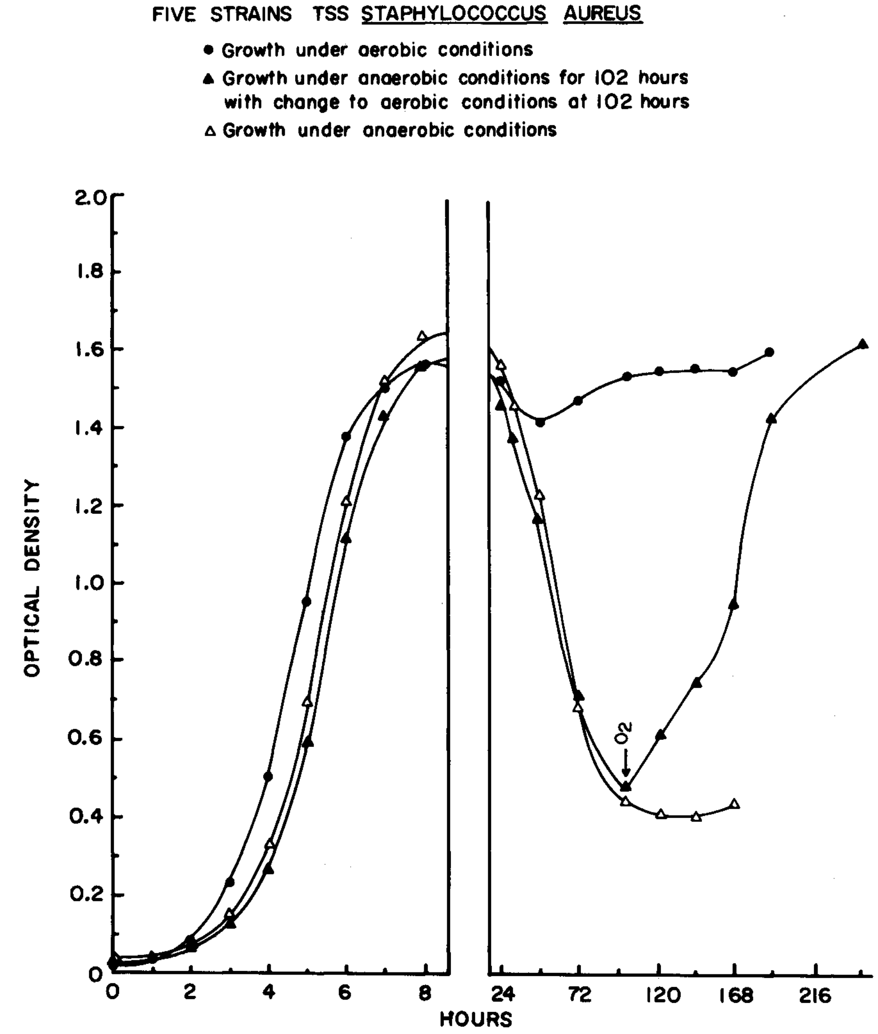

Fig. 1. The effect of air exposure on the OD of 5 TSS strains of *S. aureus* grown under various aerobic and anaerobic conditions.

microaerophilic environment into an anaerobic environment. This article reports our results in analyzing toxic shock syndrome toxin-1 (TSST-1) production in vitro under aerobic and anaerobic conditions in both logarithmic and stationary phases of growth for TSS-associated and nongenital isolates of *S. aureus* that were not associated with TSS.[9–11]

### MATERIALS AND METHODS

### Bacterial Strains

Ten strains of *S. aureus* derived from patients meeting the case criteria for the CDC were furnished by Dr. George P. Schmid of the Toxic Shock Syndrome Task Force and Dr. James C. Freely, Chief of the Special Pathogen Laboratory, CDC, Atlanta, GA. The TSST-1-producing *S. aureus* strains were supplied by Patrick M. Schlievert from the Department of Microbiology, University of Minnesota Medical School, Minneapolis. Ten nongenital strains of *S. aureus* were obtained from the Clinical Microbiology Laboratory at the University of Florida College of Medicine, Gainesville. Of these, 5 isolates were from blood cultures of different patients having septicemia; the other 5 were from patients with anaerobic staphylococcal infections who did not manifest the signs or symptoms of TSS.

### Culture Media

Stock cultures were propagated in trypticase soy broth (Baltimore Biological Laboratory, Cockeysville, MD), grown overnight, and frozen at 80°C.

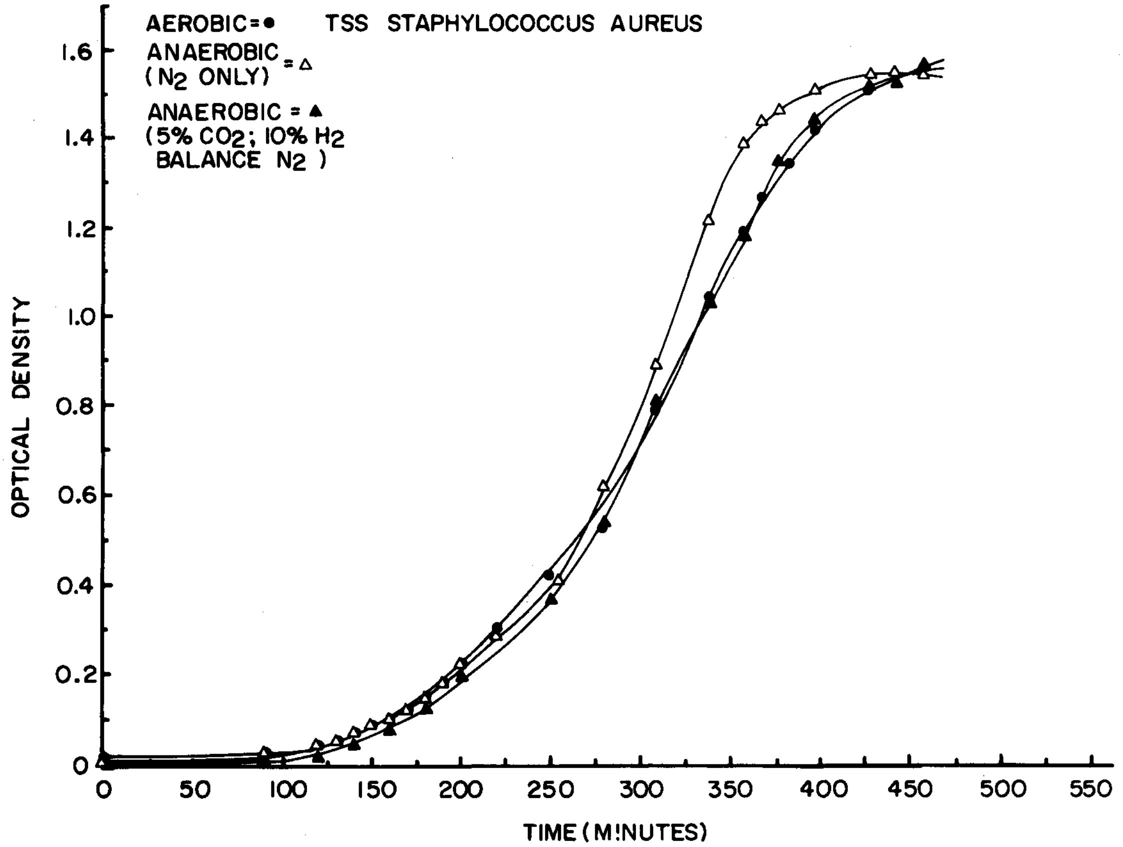

Fig. 2. The effect of aerobic and anaerobic culturing of TSS strains of *S. aureus* in terms of bacterial viability.

The liquid medium described by Johnson et al.[11] was utilized in all phases of the experiment. The medium consisted of 17 g of trypticase (Baltimore Biological Laboratory), 10 g of yeast extract (Difco Laboratories, Detroit, MI), 5 g of NaCl, and 2.5 g of $K_2$ $HPO_4$ per liter of deionized water. The pH of this medium was 7.1. The buffered medium (pH of 7.2) consisted of the above components, less the 2.5 g of $K_2$ $HPO_4$ in 0.067 M $KPO_4$ buffer. The solid medium used to determine colony-forming units (cfu) was trypticase soy agar (Difco Laboratories).

### Cell-Density Determinations

Ten different cultures of TSS strains of *S. aureus* were each grown overnight at 37°C in T-Y broth under aerobic and anaerobic conditions (gasp, Baltimore Biological Laboratory). The media to be inoculated anaerobically were prereduced in anaerobic jars for 24 h. For each strain, 4 sets of tubes containing 10 ml of T-Y broth were each inoculated with 0.1 ml of the 24-h broth culture of TSS-associated *S. aureus*. Two sets of the tubes were inoculated with a 24-h aerobic culture. Of these, 1 set was incubated aerobically and the other anaerobically. The remaining 2 sets (prereduced) were inoculated with a 24-h aerobic broth culture. One set was incubated aerobically, and the other set was incubated anaerobically. All tubes were incubated at 37°C. The optical density (OD) was measured at 52 nm using a Spectronic 100 spectrophotometer (Bausch & Lomb, Rochester, NY) over a varied time period.

### Determination of Viable Cells

Duplicate Erlenmeyer flasks, 1 containing 350 ml of T-Y broth, prereduced, were inoculated with 0.03 ml of an overnight aerobic or anaerobic starting culture of a TSS-producing strains of *S. aureus*. The flasks were incubated under aerobic or anaerobic conditions at 37°C. At varied time intervals, a 5-ml aliquot was removed from each flask. Serial 10-fold

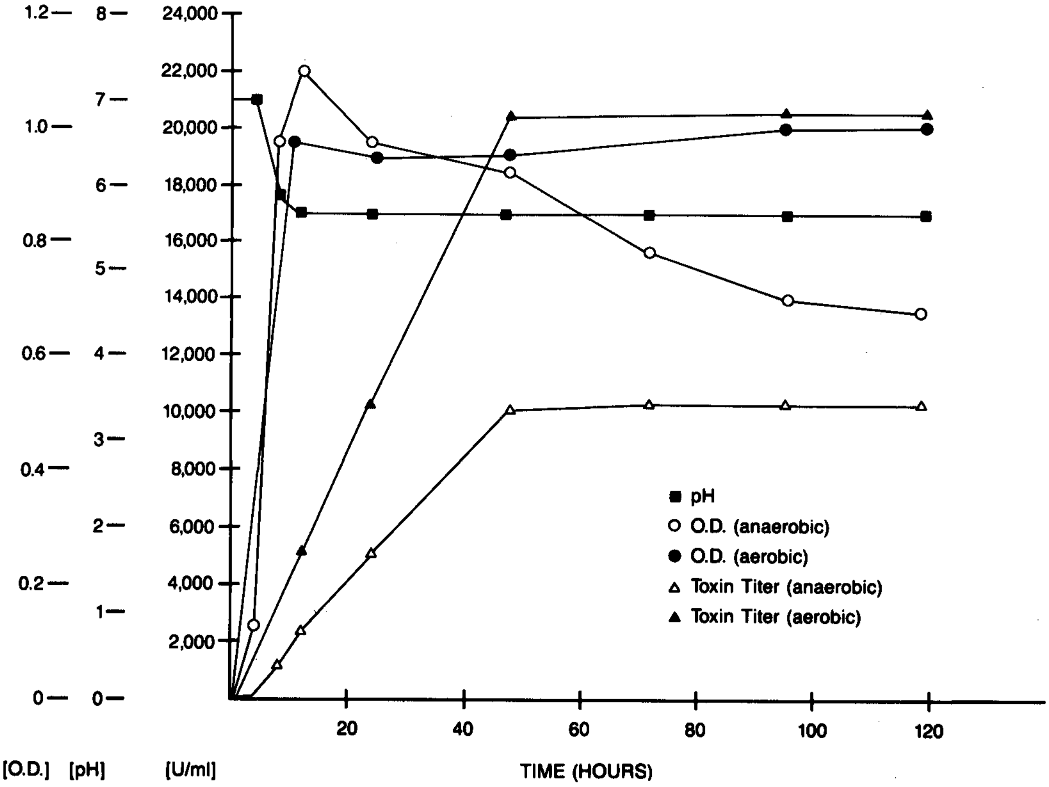

Fig. 3. Toxin production under aerobic and anaerobic culture conditions.

dilutions of each aliquot were made in sterile saline and then plated on trypticase soy agar (TSA) using the pour-late technique. The plates were incubated for 24 h at 37°C under aerobic conditions, and the colonies were counted to determine the number of colony-forming units per milliliter (cfu/ml).

### Production of TSST-1

Six sets of 8 50-ml culture tubes, each containing 30 ml of T-Y broth, were inoculated with a 0.1-ml aliquot of TSST-1-producing *S. aureus* preculture that had reached a stationary growth phase under aerobic conditions at 37°C. Three sets were incubated under aerobic conditions, and 3 sets were incubated under anaerobic conditions at 37°C. The media to be inoculated anaerobically were prereduced by boiling for 10 min, dispensed into 50-ml culture tubes, overlaid with nitrogen gas, and autoclaved at 121°C for 15 min. The inoculation of the prereduced medium was carried out in the presence of nitrogen gas. The OD (520 nm) was measured at varied time intervals. The above procedure was repeated using T-Y broth supplemented with 0.06 M phosphate buffer (see above).

### Assay of TSST-1

The toxin production was assayed using the TST-RPLA latex agglutination test (Oxoid, Inc., Columbia, MD) in microtiter plates. Briefly, the cell cultures were centrifuged at 17,000 *g* for 15 min and the supernatant filter was sterilized. Serial 2-fold dilutions of each cell-free filtrate were tested for the presence of TSST-1. The reciprocal of the highest dilution giving a positive agglutination reaction was considered the end point. The toxin titers were expressed in units per milliliter (u/ml).

### Effect of Anaerobic Growth Conditions on TSST-1 Production

Three sets of 8 25-ml culture tubes, each containing 30 ml of phosphate buffered T-Y broth, were inoculated with 0.1 ml of a TSST-1 *S. aureus* preculture. All sets were incubated at 37°C under aerobic conditions until a stationary growth phase was achieved. The stationary-phase cultures were subsequently

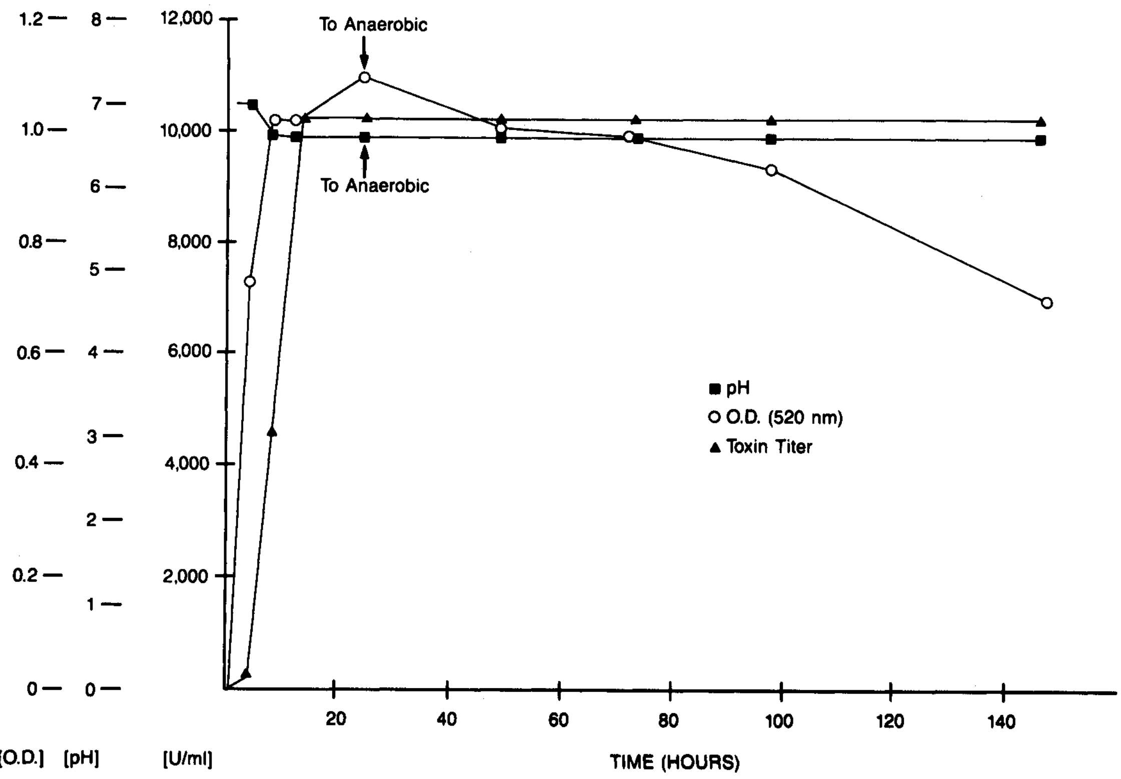

Fig. 4. The effect of anaerobic conditions on toxin production of cell cultures propagated initially under aerobic conditions: buffered media.

subjected to anaerobic conditions by placing them in Gas Pack jars. At specific times during aerobic and anaerobic culture conditions, the OD of 1 tube from each set was measured at 520 nm. The contents of each tube were subsequently centrifuged at 17,000 *g* for 10 min filter-sterilized, measured for pH, and assayed for the presence of toxin.

### Enzyme Lysis

At specific times during the culture of TSST-1, *S. aureus* enzyme lysis was performed. The pellet obtained by centrifugation of broth cultures was mixed with 1 ml of lysozyme and incubated at 37°C for 1 h. The supernatant from the above centrifugation was subsequently added to the lysed cells and this mixture was centrifuged at 17,000 *g*. The resultant preparation was then filter-sterilized and the toxin titers were measured.

### RESULTS

The aerobic and anaerobic growth curves of the strains of *S. aureus* derived from patients with in vivo evidence of TSST-1 production were analyzed. The resulting aerobic and anaerobic curves did not differ in terms of the quantity of bacteria produced during the logarithmic phase of growth or the time frame in which maximum growth was achieved (Fig. 1). While there was no initial difference quantitatively or temporally in the number of *S. aureus* colonies engendered by aerobic and anaerobic conditions, after 24 h, the curves of the bacteria grown anaerobically began to exhibit significant disparity (Fig. 1). The OD of the cultures of *S. aureus* grown under aerobic conditions remained relatively level. Those grown under strict anaerobic conditions exhibited a decrease in OD through 168 h. The fall in OD was reversed in cultures grown anaerobically when the culture was reexposed to air (Fig. 1). The quantitative assessment of viability completed by serial dilution demonstrated a 1-log reduction in the number of cfu/ml of culture medium for a given strain of *S. aureus* grown under anaerobic as opposed to aerobic conditions (Fig. 2). However, this change in the number of viable bacteria did not occur until 50–60 h. The changes in the OD observed were not unique to the TSS stains of *S. aureus*. Comparable changes were observed with the nongenital strains of *S. aureus*.

### TSST-1 Production

The elaboration of TSST-1 by toxigenic strains of *S. aureus* occurred during the logarithmic phase of cell growth under aerobic as well as under anaerobic conditions. The peak toxin titers occurred after 48 h of incubation (Fig. 3) and remained constant for 96 h under both aerobic and anaerobic conditions. Despite a fall in the OD of the TSS culture grown anaerobically, the toxin production plateaued and remained at a constant level. The extension of anaerobic conditions resulted in no further increase in toxin production, despite a further fall in OD. In this study, a clear 2-fold-higher toxin titer was obtained under aerobic conditions. An exposure of the stationary-phase aerobic cultures to anaerobic culture conditions revealed that peak toxin titers occurred at 24 h of incubation with no additional toxin production noted under prolonged anaerobic conditions (Fig. 4). The enzyme lysis of cell cultures resulted in no appreciable increase in toxin titer. The effect of pH on toxin titer was negligible, as unbuffered media with a pH range of 5.5–7.0 and buffered media with a pH range of 6.7–7.1 exhibited similar peak toxin titers.(Fig. 4).

### DISCUSSION

The appearance of a large number of cases of TSS coincided with a significant technological advance in tampon manufacturing. The documentation of the existence of TSS-producing strains of *S. aureus* antedated the introduction of superabsorbent tampons. In some way, the superabsorbent tampons appeared to produce a change which took the physiologic effects of nonenteric exotoxin from subclinical to clinical levels. Two possible mechanisms were postulated: increased absorbency resulting in augmented substrate availability or vaginal occlusion due to volumetric expansion in tampon size transforming a microaerophilic environment into one capable of sustaining obligatory anaerobic replication.

The staphylococci are the only genus of bacteria that is able to function both as class-I anaerobes (bacteria that replicate better in the presence of air than they do in its absence) and class-III anaerobes (bacteria that are destroyed by even transient exposure to molecular oxygen). In this study, the strict anaerobic conditions did not influence the initial quantity of cfu/ml engendered in the logarithmic phase of growth. Even though, after 24 h, the cultures grown anaerobically exhibited a progressive fall in OD, the peak quantity of viable bacteria present in the stationary phase of growth remained constant for 50 h.

This report infers that a potential conversion of the endocervical or vaginal area from a microaerophilic environment to an anaerobic environment would not enhance TSST-1 production. Cell rupture did not result in an increase detectable TSST-1 titers. TSST-1 is produced primarily by TSS strains of *S. aureus* during the logarithmic phase of growth.[12] The maximum titers are achieved under aerobic growth conditions. Once the stationary phase of growth is reached with prolonged anaerobic conditions, the production of TSST-1 toxin is reduced. This in vitro observation correlates well with in vivo clinical data. Clinically, the majority of TSS cases occur early in the menstrual cycle (within 48 h). With wounds, an infection reflecting the onset of signs and symptoms usually occurs on the fourth day.[13–16] In those cases of TSS associated with *S. aureus* wound infections, the TSS usually begins as early as the second day at a time when the local signs of wound infection are minimal or absent. The determinants of TSS appear to be 1) *S. aureus* strain selection and 2) augmental bacterial replication for growth that functions in concert. The possibility that superabsorbent tampons promote the probability of TSS by an augmentation of the available substrate for TSS strains of *S. aureus* requires further experimental analysis.

### ACKNOWLEDGMENT

The authors thank Dr. Murray Joseph Casey for his review and editorial assistance with this manuscript.

### REFERENCES

1. Centers for Disease Control and Prevention: Toxic shock syndrome—United States. MMWR 29:229–230, 1980.
2. Centers for Disease Control and Prevention: Follow-up on toxic shock syndrome—United States. MMWR. 29:297–299, 1980.
3. Centers for Disease Control and Prevention: Follow-up on toxic shock syndrome. MMWR 29:441–445, 1980.
4. Centers for Disease Control and Prevention: Toxic shock syndrome. MMWR 29:495, 1980.
5. Schmid GP, Shands KN, Dana BB: Toxic shock syndrome. Infect Dis Lett Obstet Gynecol 3:1–6, 1980.

6. Shands KN, Schmid GP, Dan BB, et al.: Toxic shock syndrome in menstruating women: Association with tampon use and *Staphylococcus aureus* and clinical features in 52 cases. N Engl J Med 303:1436–1442, 1980.
7. McKenna UG, Meadows JA III, Brewer MS, et al.: Toxic shock syndrome, a newly recognized disease entity: Report of 11 cases. Mayo Clinic Proc 55:663–672, 1980.
8. David JP, Chesney PJ, Want PJ, et al.: Toxic shock syndrome: Epidemiologic features, recurrence, risk factors, and prevention. N Engl J Med 303:1429–1435, 1980.
9. Schlievert PM, Shands KN, Dan BB, Schmid GP, Mishimura RD: Identification and characterization of an exotoxin from *Staphylococcus aureus* associated with toxic shock syndrome. J Infect Dis 143:509–516, 1981.
10. Bergdoll MS, Reiser RF, Crass BA, Robbins RN, Davis JP: A new staphylococcal enterotoxin, enterotoxin F associated with toxic shock syndrome *Staphylococcus aureus* isolates. Lancet 1:1017–2110, 1981.
11. Johnson A, Metzger JF, Spero L: Production, purification and chemical characterization of *Staphylococcus aureus* exfoliative toxin. Infect Immun 12:1206–1210, 1975.
12. Schlievert PM, Blomster DA: Production of staphylococcal pyrogenic exotoxin type C: Influence of physical and chemical factors. J Infect Dis 147:236–242, 1983.
13. Doran KJ, Thompson DM, Conn AR, Whitlmann BK, Stiver HG, Chow AW: Toxic shock syndrome in the postoperative patient. Surg Gynecol Obstet 154:65–68, 1982.
14. Ishaki KG, Rogers WA: Cryptogenic acute cholangitis: Association with toxic shock syndrome. Am J Clin Pathol 76:619–626, 1981.
15. Schlievert PM. Alteration of immune function by staphylococcal pyrogenic exotoxin C: Possible role in toxic shock syndrome. J Infect Dis 147:391–398, 1981.
16. Schlievert PM, Osterholm T, Kelly JA, Nishimura RD: Toxin and enzyme characterization of *Staphylococcus aureus* isolates from patients with and without toxic shock syndrome. Ann Intern Med 96:937–940, 1982.
